# Milk fermented with *Lactobacillus rhamnosus* R0011 induces a regulatory cytokine profile in LPS-challenged U937 and THP-1 macrophages

**DOI:** 10.1016/j.crfs.2020.02.002

**Published:** 2020-02-26

**Authors:** Michael P. Jeffrey, Holly Jones Taggart, Janice L. Strap, Gibran Edun, Julia M. Green-Johnson

**Affiliations:** a Applied Bioscience Graduate Program, Ontario Technical University, Oshawa, ON, Canada; b Faculty of Health Sciences, Ontario Technical University, Oshawa, ON, L1G 0C5, Canada; c Faculty of Science, Ontario Technical University, Oshawa, ON, L1G 0C5, Canada

**Keywords:** Fermented milk, Macrophage polarization, Bioactives, Lactobacilli

## Abstract

Fermented dairy products have become attractive functional foods for the delivery of probiotics and their biologically active metabolites. The aim of this study was to examine the immunomodulatory activity of milk fermented with the probiotic lactic acid bacterium *Lactobacillus rhamnosus* R0011 (LrF) on macrophages challenged with lipopolysaccharide (LPS), a potent pro-inflammatory stimulus. To this end, human THP-1 or U937 monocytes were differentiated into resting macrophages then stimulated with LPS and co-incubated with the LrF or with milk controls. Levels of pro-inflammatory and immunoregulatory cytokines were determined by enzyme-linked immunosorbent assays. Culturing of LPS-stimulated U937 macrophages with either the whole or filtered LrF resulted in an increase in Interleukin (IL)-1Ra production relative to the negative control. THP-1 macrophages cultured with the LrF demonstrated an increase in LPS-induced IL-10 and IL-1β production, while production of LPS-induced IL-6, sCD54, IL-8, IL-1β, TNF-α, IL-12p70 and Transforming Growth Factor-β (TGF-β) was unaffected. Further, the LrF induced the expression of *DC-SIGN* and *CD206*, markers of immunoregulatory M2 macrophage polarization, in LPS-challenged THP-1 macrophages. Taken together, milk fermented with *L. rhamnosus* R0011 increased regulatory cytokine production from LPS-challenged U937 and THP-1 macrophages, while simultaneously up-regulating the production of IL-1β and expression of *DC-SIGN* and *CD206*, a profile characteristic of polarization into the immunoregulatory M2 macrophage phenotype.

## Highlights

• Milk fermented with *Lactobacillus rhamnosus* R0011 (Lrf) induces a regulatory macrophage phenotype.
• Modulation of cytokine profiles induced by lipopolysaccharide challenge consistent with an alternatively activated (M2) macrophage phenotype.
• Fermented milk conditioning induced macrophage expression of genes characteristic of M2 macrophage polarization (*DC-SIGN* and *CD206*).

## Graphical abstract

Image created with Biorender.

**Figure:**
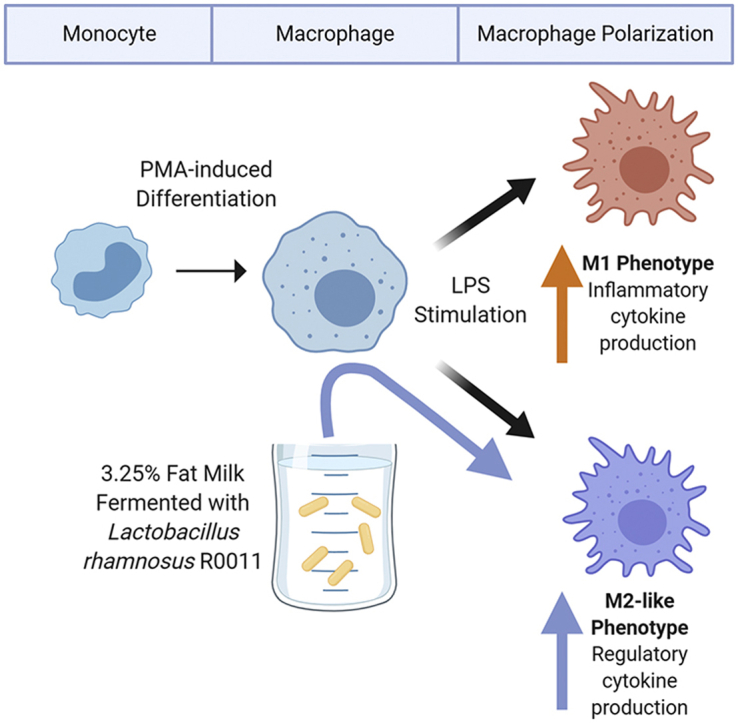

## 1 Introduction

Dairy is an established component of the North American diet with fermented dairy products becoming popular functional foods for the delivery of probiotics and their biologically active metabolites. Proposed health-related effects of consuming fermented milk include modulation of the host gut microbiota, down-regulation of pro-inflammatory biomarkers, antioxidant activity, alleviation of food allergies and sensitivities (including improved lactose tolerance), alleviation of antibiotic-induced diarrhea, and antagonism of gut-associated pathogens (Bordoni et al., 2017, Astrup, 2014, Parvez et al., 2006, Wagar et al., 2009, Virtanen et al., 2007). Dairy products containing probiotic lactic acid bacteria have been reported to change the expression of microbiome-encoded enzymes with downstream effects seen in many metabolic pathways (McNulty et al., 2011). Recently, fermented milk consumption has been shown to alter postprandial serum metabolite composition, metabolic and inflammation-associated whole blood transcriptome profiles, and insulin responses in healthy men (Pimentel et al., 2018, Burton et al., 2018), suggesting the potential for systemic immune and metabolic impacts of bioactive components within fermented dairy products.

While fermented dairy and soy milk impact on host intestinal epithelial cells has been demonstrated (Wagar et al., 2009, Lin et al., 2016), many questions remain about the potential of fermented milks to modulate systemic host immune activity through contact with cell types participating in innate and adaptive immunity. Macrophages, key phagocytic cellular players in the host immune response, detect bacterial components in their environment through the recognition of microbe-associated molecular patterns (MAMPs) via pattern recognition receptors such as the Toll-like receptors (TLRs). Tissue macrophages are a heterogeneous population harbouring the M1 and M2 phenotypes (Legein et al., 2013). M1 macrophages, also known as classically activated macrophages, are induced by TLR recognition of conserved MAMPs such as lipopolysaccharides (LPS). Once activated, M1 macrophages produce inflammatory cytokines including tumour necrosis factor-α (TNF-α), interleukin-6 (IL-6), IL-1β, and interleukin-12 (IL-12) (Moore and Tabas, 2011). In contrast, M2 macrophages, or alternatively activated macrophages, produce regulatory cytokines such as interleukin-10 (IL-10), interleukin-1 receptor antagonist (IL-1Ra), and TGF-β and mediate tissue repair through collagen secretion (Legein et al., 2013; Moore et al., 2013). Recent evidence suggests that certain probiotic and commensal bacteria can modulate macrophage phenotype and activity. For example, a commercially available probiotic multi-strain supplement (VSL#3) alters cellular morphology and cytokine production profiles in polarized M1 and M2 macrophages (Isidro et al., 2014), and several probiotic strains increase LPS-induced production of TNFα by M1 macrophages (Habil et al., 2011). The cell-free supernatant of milk fermented with *Lactobacillus paracasei* FT700 was shown to induce differentiation of monocytes into macrophages (Tulini et al., 2015), suggesting a novel role of bioactive fermentation products on host immune outcomes that warrants further study.

In this study, the impact of dairy milk-cultured *L. rhamnosus* R0011 on macrophage cytokine production profiles was examined. Secretome derived from *L. rhamnosus* R0011 down-regulates inflammatory mediator production from challenged intestinal epithelial cells, suggesting a role for bioactive molecule production by this lactic acid bacterium (Jeffrey et al., 2018). When cultured in milk, *L. rhamnosus* R0011 produces soluble mediators that down-regulate interleukin (IL)-1β-induced prostaglandin production, a key lipid mediator in the inflammatory response, from human intestinal epithelial cells (IEC) (Fiander et al., 2005). However, the impact of *L. rhamnosus* R0011-derived fermented milk on monocytes and macrophages, key participants in the immune response, is currently unknown. Hence, we examined the effects of milk fermented with *L. rhamnosus* R0011 on differentiated THP-1 and U937-derived macrophages stimulated *in vitro* with the TLR ligand LPS to determine the impact on cytokine production. The THP-1 cell line is a well-established model for studying the roles of both monocytes and macrophages in inflammation *in vitro* (Qin, 2012, Chanput et al., 2014). Stimulation of THP-1 and U937 cells with LPS elicits cytokine expression profiles similar to those observed in monocytes and macrophages *in vivo* (Sharif et al., 2007). THP-1 and U937 monocytes can be differentiated into macrophages with the addition of phorbol-12-myristate-13-acetate (PMA; Chanput et al., 2014) and all-trans retinoic acid (ATRA; Masotti et al., 2011) respectively, providing a useful *in vitro* approach to examine the effects of milk fermentation on macrophage phenotypes.

## 2 Materials and methods

### 2.1 Preparation of the LrF

Commercially available pasteurized whole milk (3.25% milk fat) was inoculated with 1 × 10^8^ CFU/mL of *L. rhamnosus* R0011 (provided by Lallemand Health Solutions, Montreal, QC, Canada) and incubated at 37 °C with agitation (220 rpm) for approximately 28 h until a pH of 4.5 ± 0.1 was obtained. A non-fermented milk control and a lactic acid acidified milk control were also subjected to the same incubation period under the same conditions. A portion of the *L. rhamnosus* R0011 ferment was filtered through Millex – SV 5.0 μm (PVDF membrane; Millipore Sigma, MA, USA), 1.2 μm (cellulose acetate membrane; Sterlitech, WA, USA), and 0.2 μm (PES membrane; FroggaBio, ON, CA) filters to remove the bacteria and used to determine whether or not any observed immunomodulatory activity could be attributed to bacterial fermentation products. The lactic acid acidified milk control was prepared by determining the concentration of lactic acid in the *L. rhamnosus* R0011 ferment (LrF) using the Megazyme Lactic Acid Determination kit (Wicklow, Ireland) and adding the determined amount of L-lactic acid to the acidified control. If further reduction in pH was necessary following the addition of the correct concentration of lactic acid, concentrated HCl was added to reduce the pH of the acidified control to that of LrF. *L. rhamnosus* R0011 was enumerated following fermentation by plating serial dilutions on DeMan Rogosa Sharpe (MRS; BD Diagnostic Systems, Sparks, MD, USA) agar plates.

### 2.2 Cell culture

The THP-1 human peripheral blood monocyte cell line (ATCC #TIB-202) was maintained in RPMI-1640 medium supplemented with 0.05 mM β-mercaptoethanol, 10% fetal bovine serum (FBS) and 0.05 mg/mL gentamicin in a humidified incubator at 37 °C and 5% CO_2_. THP-1 cells were sub-cultured every 4–5 days. The U937 human myeloid monocyte cell line (ATCC #CRL-1593.2) was maintained in RPMI-1640 supplemented with 10% FBS, 1 mM sodium pyruvate, 2 mM L-glutamate and 0.05 mg/mL gentamicin. Cells were cultured similarly to the THP-1 cells.

### 2.3 Differentiation of THP-1 monocyte cells into resting macrophages

Differentiation of THP-1 monocyte cells into resting Mθ macrophages was conducted as previously described by Chanput et al. (2013). THP-1 cells were seeded in 12-well tissue culture plates (CellStar, Greiner Bio-One, NC, USA) at a concentration of 1 × 10^6^ cells/mL. These cells were treated with 100 ng/mL of phorbol-12-myristate-13- acetate (PMA) at 37 °C, 5% CO_2_ in a humidified incubator. After a 48 h-incubation, the PMA-containing culture medium was removed and cells were washed twice with complete THP-1 medium and allowed to rest in complete medium for an additional 24 h prior to challenge. Differentiation was confirmed by immunophenotyping for increased expression of the macrophage cell surface marker CD11b.

### 2.4 Differentiation of U937 human myeloid monocyte cells into resting macrophages

U937 cells were seeded at a concentration of 1 × 10^6^ cells/mL in 12-well tissue culture plates (CellStar, Greiner Bio-One, NC, USA) using non-supplemented RPMI-1640 medium. Immediately after seeding, U937 cells were differentiated to the monocyte stage using 1 μM all-trans retinoic acid (ATRA) for 24 h at 37 °C under 5% CO_2_ in a humidified incubator, as previously described (Zhang et al., 2008). Medium containing ATRA was aspirated following incubation prior to cell challenge. Differentiation was confirmed by immunophenotyping for increased expression of the macrophage cell surface marker CD11b.

### 2.5 THP-1 and U937 cell challenge with lipopolysaccharide and conditioning with LrF

U937 and THP-1 macrophages at a concentration of 5 × 10^5^ cells/mL in RPMI-1640 medium supplemented with 10% FBS were challenged with LPS (125 ng/mL) and cultured for 24 h with LrF (either whole or filtered) or lactic acid acidified controls (either whole or filtered) or an unfermented milk control. The cells were then collected by centrifugation at 300×*g* for 10 min and supernatants were stored at −80 °C pending cytokine analysis. Cell viability was determined by either the XTT (2,3-Bis-(2-Methoxy-4-Nitro-5-Sulfophenyl)-2H-Tetrazolium-5-Carboxanilide) (Sigma-Aldrich, MO, USA) cell viability assay (Roehm et al., 1991) or the Trypan Blue (Sigma-Aldrich, MO, USA) exclusion assay. Initial characterization of the effects of the LrF or milk controls was conducted by testing serial dilutions (1/10, 1/50, 1/100, 1/1000) of the ferments and acidified or non-acidified controls on the cells. All subsequent challenges only examined the effects of the 1/50 LrF dilution (and pH-matched controls) as this concentration was shown to modulate the immune response without negatively impacting cell viability.

### 2.6 Cytokine quantification

Cytokines were quantified from cell culture supernatants using enzyme-linked immunosorbant assays (ELISA) following manufacturer's protocols (R&D Systems, MN, USA), including human IL-1β (Cat. no DY201), IL-6 (Cat. no DY206), IL-1Ra (Cat. no DY280), IL-8 (Cat. no DY208), TNF-α (Cat. no DY210), and TGF-β (Cat. no DY208). ELISA for IL-10 (Cat. no 430603) and IL-12(p70) (Cat. no 431702) using cell culture supernatants were also performed following manufacturer's protocols (BioLegend, CA, USA). All ELISAs were done using 96-well high-binding Microlon 600 ELISA plates (Greiner Bio-One, NC, USA) and plates were read at a wavelength of 450 nm using a Synergy HTTR microplate reader (Bio-Tek Instrumentation, VT, USA).

### 2.7 Determination of cell surface molecule expression

Cell surface molecule expression was quantified using the Millipore Guava Personal Cell Analysis (PCA) System (Millipore Sigma, MA, USA). Following LPS challenge, differentiated U937 and THP-1 macrophages were dislodged from the tissue culture plates using a cell scraper, and collected by centrifugation at 300×*g* for 10 min. Viable cells were counted using the Trypan Blue exclusion assay and resuspended at a concentration of 1 × 10^6^ cells/mL before staining for 30 min on ice in the dark with phycoerythrin (PE)-conjugated antibodies and their corresponding isotype controls (BioLegend, CA, USA). Antibodies used were human anti-CD11b (Clone ICRF44) and IgG1, κ (Clone MOPC-21). Data were collected using the Guava Express Software (Version 6.0.2). All data were reported as mean fluorescence intensity (MFI). FCS Express Flow Cytometry Software (Version 3.0) was used for generation of histograms and the Overton subtraction was used to determine percentage positive CD11b expression.

### 2.8 Comparative RT-qPCR

RT-qPCR to examine M2 macrophage specific marker expression was performed as per the MIQE guidelines. Total RNA was harvested using the TRIzol method (Chomczynski, 1993) following manufacturer's protocols(ThermoFisher Scientific, MA, USA) and purified using the RNeasy Plus Mini Kit (Qiagen, Hildon, Germany). DNase-treated RNA(1 μg) from controls and each challenge were reverse transcribed with Superscript IV following manufacturer's protocols as previously described (Macpherson et al., 2014). Reverse-transcribed cDNA was diluted 1:4 prior to amplification and 2.5 μl of diluted cDNA was used in RT-qPCR using gene-specific primers (Table S1) (Chen et al., 2015, Locati et al., 2002, Maess et al., 2010, Zarember and Godowski, 2002) and SsoFast EvaGreen Supermix (Bio-Rad, CA, USA) per the manufacturer's instructions. An initial incubation of 5 min at 95 °C was performed, followed by 40 cycles consisting of template denaturation (15 s at 95 °C) and one-step annealing and elongation (30 s at 60 °C), with a Bio-Rad CFX Connect instrument (Bio-Rad, CA, USA). Three biological replicates were analyzed for each gene tested, and fold change expression levels were normalized to the expression levels of two reference genes(*RPL37A* and *ACTB*) and negative controls using Bio-Rad CFX Manager 3.1 software.

### 2.9 Statistical analyses

Statistical analyses were done using the GraphPad Prism software version 8 (GraphPad Software, CA, USA), and included one-way analysis of variance (ANOVA) and Tukey's multiple comparison test for significant differences (P < 0.05). For some analyses, the Student's paired *t*-test was used for two-group comparisons. All data are shown as the mean ± standard error of the mean (SEM) where one biological replicate (n = 1) is representative of three technical replicates; for most conditions, n = 3 or greater.

## 3 Results

### 3.1 Conditioning U937 or THP-1 human monocyte cells with LrF and LPS has differential effects on cytokine production

U937 human monocyte cells were cultured with the LrF and the production of IL-8, sCD54, and IL-1Ra was determined. There was no change in IL-8 (Fig. 1A) or IL-1Ra (Fig. 1C) production between LPS-challenged monocytes and those stimulated with LPS and conditioned with the LrF or milk controls. However, there was a significant down-regulation of sCD54 production by monocytes cultured with the whole LrF. This downregulation was also observed for U937 monocytes cultured with the lactic acid and non-fermented milk controls (Fig. 1B), suggesting an effect of a milk component not associated with fermentation or acidification. In contrast, THP-1 human monocytes cultured in media containing the *L. rhamnosus* R0011-derived milk ferments or milk controls showed no changes in LPS-induced IL-8 (Fig. 1D), sCD54 (Fig. 1E), or IL-1Ra production (Fig. 1F).

**Fig. 1:**
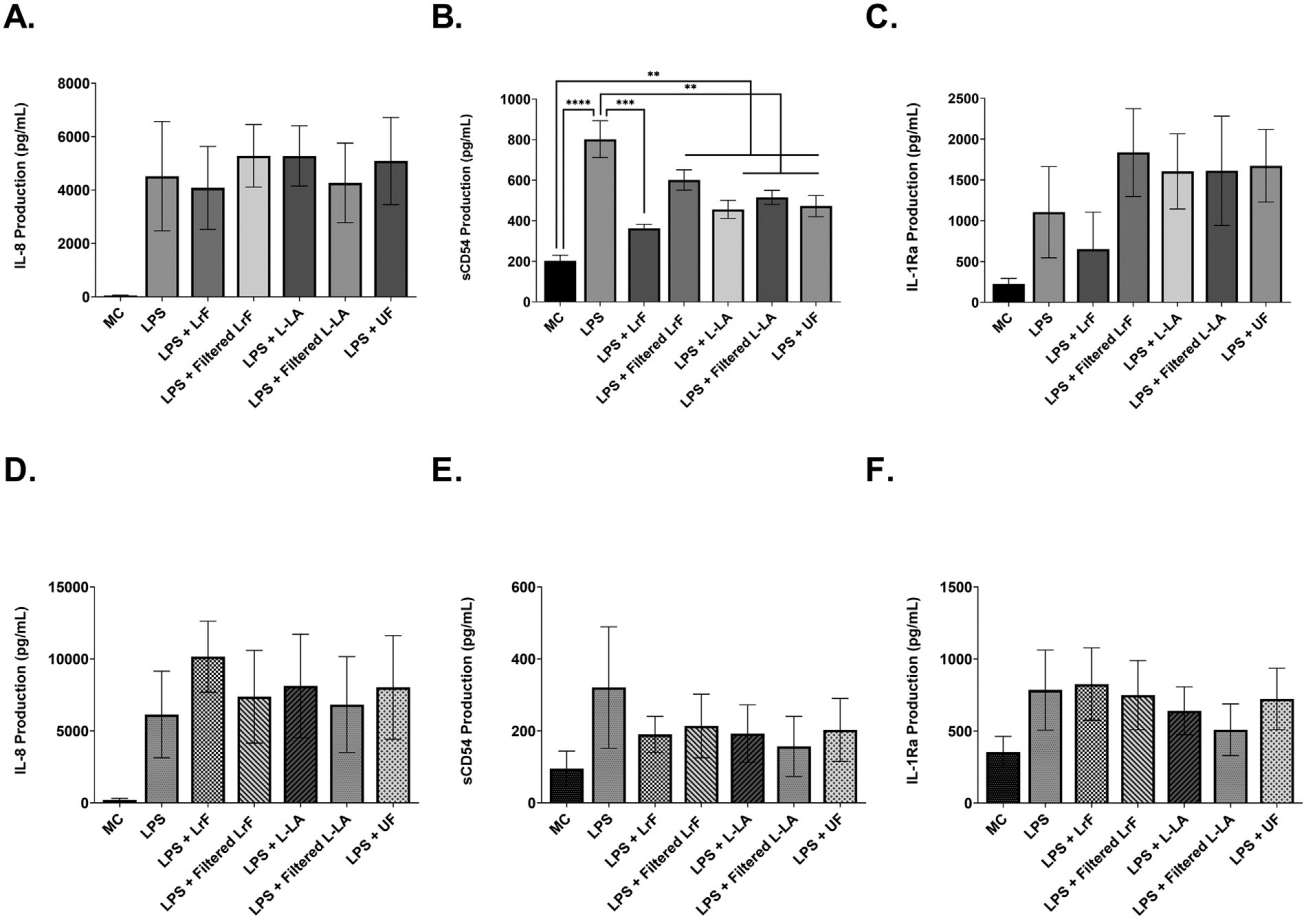
U937 and THP-1 monocytes stimulated with 125 ng/mL LPS and incubated with *L. rhamnosus* R0011 whole/filtered ferments or milk controls concurrently for 24 h (n = 3) **A.** Mean IL-8 production from U937 monocytes ± SEM **B.** Mean sCD54 production ± SEM from U937 monocytes **C.** Mean IL-1Ra production from U937 monocytes ± SEM **D.** Mean IL-8 production from THP-1 monocytes ± SEM **E.** Mean sCD54 production ± SEM from THP-1 monocytes and **F.** Mean IL-1Ra production from THP-1 monocytes ± SEM. Cell viability was >80% for all treatments, as determined by XTT and Trypan Blue Exclusion Viability assays. Significance between treatment groups as determined by Tukey's one-way ANOVA is indicated as ∗ (P < 0.05), ∗∗ (P < 0.01), ∗∗∗ (P < 0.001), or ∗∗∗∗(P < 0.0001).

### 3.2 Conditioning U937-derived macrophages with the LrF and LPS results in an increase in IL-10 and IL-1Ra production

U937-derived resting macrophages were cultured with the LrF or milk controls and LPS and changes in IL-1β, IL-10, IL-1Ra, IL-8, IL-6 were measured. There was a significant increase in LPS-induced IL-10 production by macrophages cultured with the LrF whole milk ferment relative to LPS-challenged macrophages cultured with the whole lactic acid control or the unfermented milk control (Fig. 2B). There was also a significant increase in LPS-induced IL-1Ra production by macrophages cultured with both the whole and filtered LrF or the unfermented milk controls relative to the negative control (Fig. 3C). Finally, there was a significant increase in LPS-induced IL-6 production by macrophages cultured with the whole LrF. This increase in LPS-induced IL-6 production was not observed for macrophages incubated with the lactic acid or non-fermented milk controls (Fig. 3E). In contrast, there was no modulation of LPS-induced IL-8 (Fig. 2D), IL-1β (Fig. 2A), sCD54 production (Supplemental Figure 1A), total TGF-β production (Supplemental Figure 1B), IL-12p70 (data not shown) or TNF-α production (data not shown) between U937-derived macrophages challenged with LPS or those cultured with LrF or controls and LPS.

**Fig. 2:**
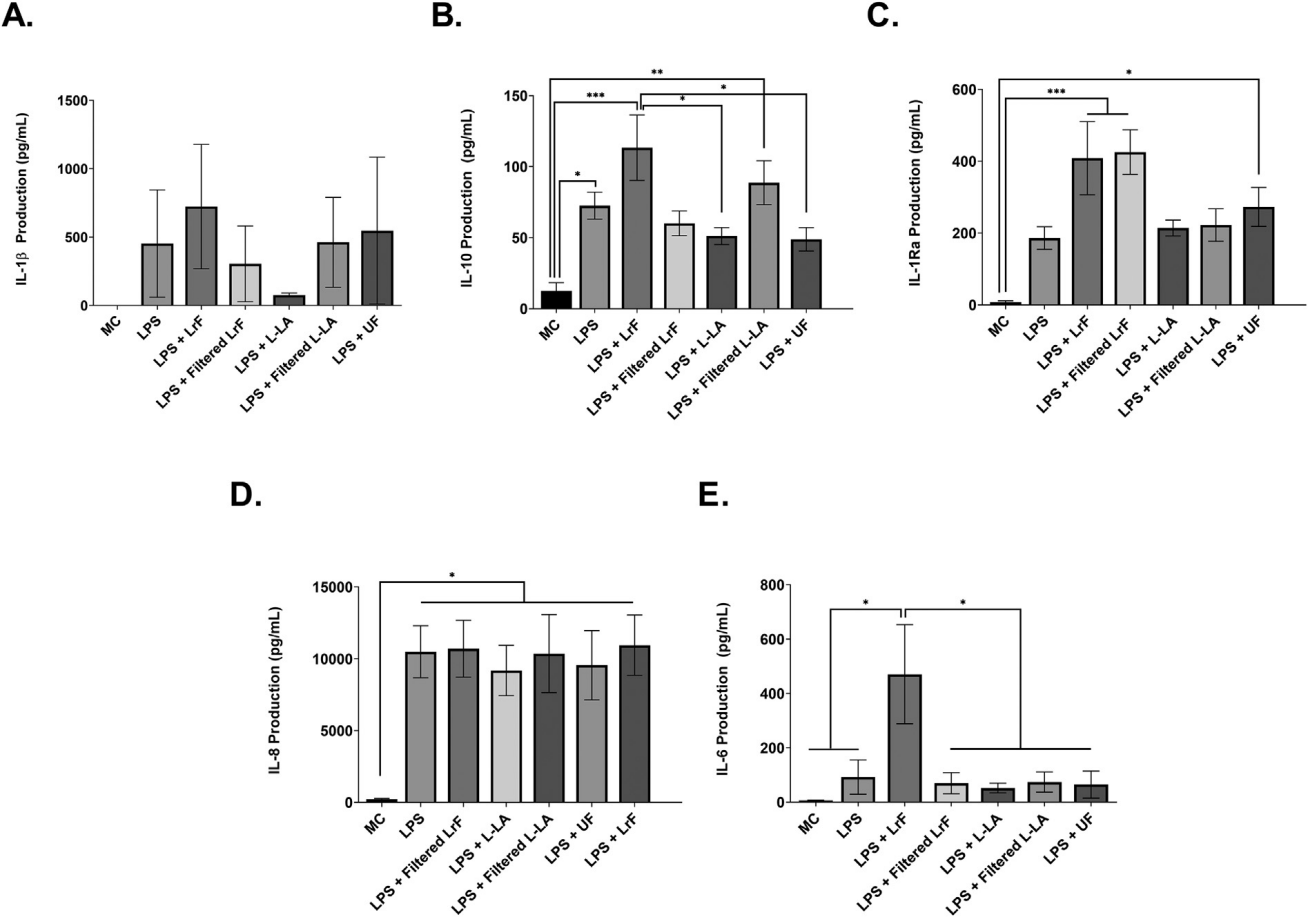
U937-derived Mθ macrophages stimulated with 125 ng/mL LPS and incubated with *L. rhamnosus* R0011 whole/filtered ferments or milk controls concurrently for 24 h (n = 3–5) **A.** Mean IL-1β production ± SEM **B.** Mean IL-10 production ± SEM **C.** Mean IL-1Ra production ± SEM **D.** Mean IL-8 production ± SEM and **E.** Mean IL-6 production ± SEM. Cell viability was >90% for all treatments, as determined by the Trypan Blue Exclusion Viability assays. Significance between treatment groups as determined by Tukey's one-way ANOVA is indicated as ∗ (P < 0.05), ∗∗ (P < 0.01), ∗∗∗ (P < 0.001), or ∗∗∗∗(P < 0.0001).

**Fig. 3:**
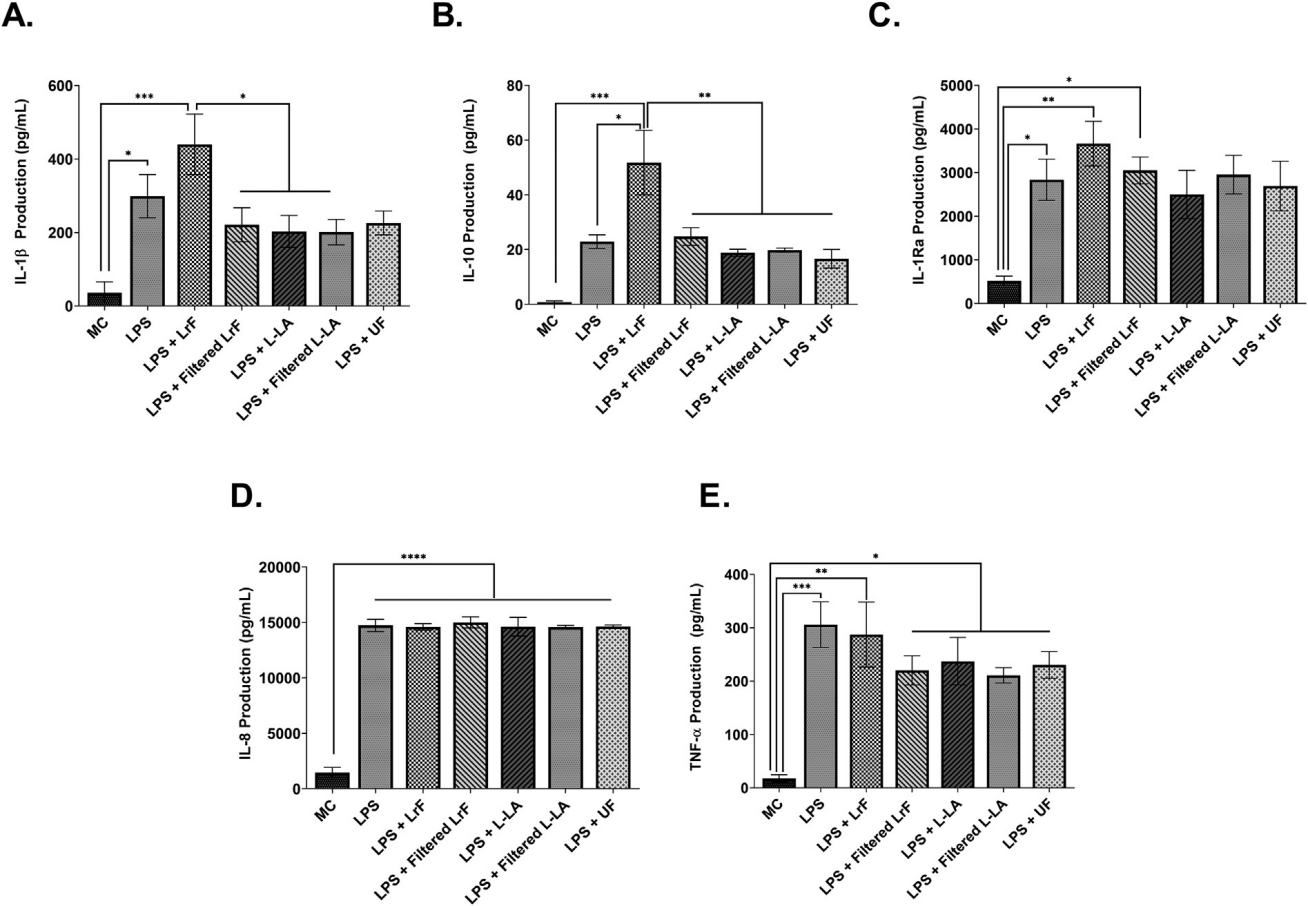
THP-1-derived Mθ macrophages stimulated with 125 ng/mL LPS and incubated with *L. rhamnosus* R0011 whole/filtered ferments or milk controls concurrently for 24 h (n = 3–5) **A.** Mean IL-1β production ± SEM **B.** Mean IL-10 production ± SEM **C.** Mean IL-1Ra production ± SEM **D.** Mean IL-8 production ± SEM and **E.** Mean TNF-α production ± SEM. Cell viability was >90% for all treatments, as determined by the Trypan Blue Exclusion Viability assays. Significance between treatment groups as determined by Tukey's one-way ANOVA is indicated as ∗ (P < 0.05), ∗∗ (P < 0.01), ∗∗∗ (P < 0.001), or ∗∗∗∗(P < 0.0001).

### 3.3 Conditioning THP-1-derived macrophages with LrF and LPS results in an increase in IL-10 and IL-1β

**Fig. 4:**
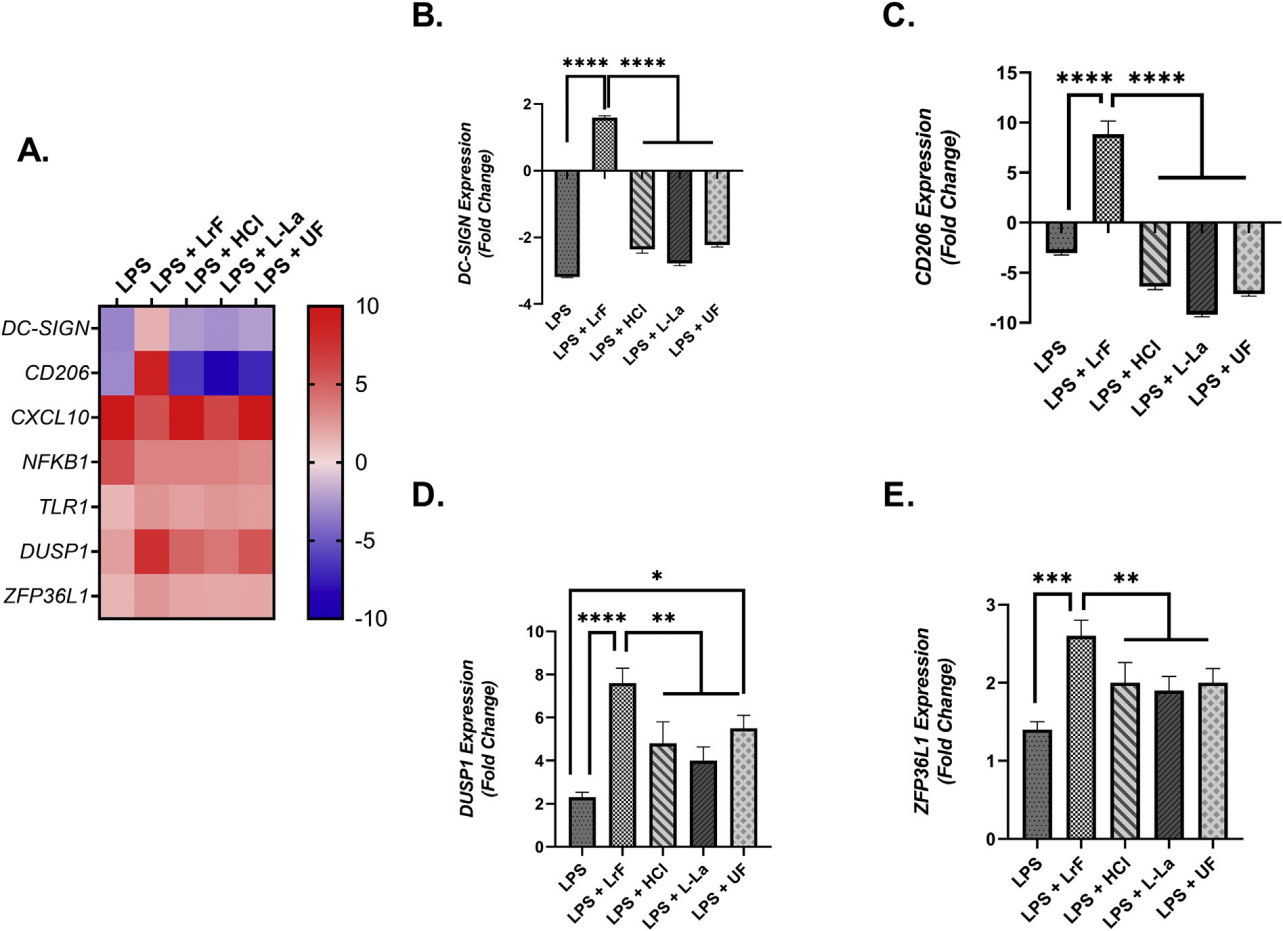
Differential gene expression profiles of THP-1-derived Mθ macrophages stimulated with 125 ng/mL LPS and incubated with *L. rhamnosus* R0011 whole ferments or milk controls concurrently for 24 h (n = 3). Significance between treatment groups as determined by Tukey's one-way ANOVA is indicated as ∗ (P < 0.05), ∗∗ (P < 0.01), ∗∗∗ (P < 0.001), or ∗∗∗∗(P < 0.0001).

THP-1 resting macrophages were cultured with LPS and the *L. rhamnosus* R0011-derived milk ferments or milk controls and changes in IL-1β, IL-10, IL-1Ra, IL-8, IL-6, sCD54, IL12-p70 and total TGF-β were measured. IL-10 production was significantly increased for macrophages cultured with LPS and the LrF. This increase was not seen for THP-1-derived macrophages cultured with the acidified or non-fermented milk controls (Fig. 3B). There was also a significant increase in IL-1β production by macrophages cultured with LPS and the LrF. This increase was not observed in cells cultured with the acidified or non-fermented milk controls (Fig. 4A). In contrast, there was no change in LPS-induced IL-1Ra (Fig. 3C), IL-8 (Fig. 3D), TNF-α (Fig. 3E), total TGF-β (Supplemental Figure 1A), IL-6 (Supplemental Figure 1B), IL-12p70 production (Data not shown).

### 3.4 Conditioning of LPS-challenged THP-1-derived macrophages with the LrF induces transcriptional changes consistent with M2 immunoregulatory macrophage polarization

To further investigate the impacts of the LrF on LPS-challenged THP-1-derived macrophages, changes in the expression of genes associated with M2 immunoregulatory macrophage polarization were examined. LPS-induced *CXCL10* expression, a potent pro-inflammatory chemokine, and *NFKB1*, a central regulator of the innate immune response, were reduced by the LrF. This effect was also seen in acidified and unfermented milk controls (Supplemental Figure 2). However, expression of *DC-SIGN*, *CD206*, *DUSP1*, and *ZFP36L1*, M2 macrophage-associated polarization markers, was significantly higher in LPS-challenged THP-1-derived macrophages co-challenged with the LrF, an effect which was not seen in cells cultured with the acidified or unfermented milk controls (Fig. 4).

## 4 Discussion

Fermentation modifies milk components, producing a range of bioactive components including immunomodulatory peptides and immunoregulatory tryptophan and glutamate derivatives (Pessione and Cirrincione, 2016, Yilmaz and Gokmen, 2018, Griffiths and Tellez, 2013, Bertazzo et al., 2016, Fernandez et al., 2017, Fernandez and Marette, 2018). While fermented dairy products are attractive as functional foods, many questions remain regarding their modes of action in the context of health and impact on the immune system. For example, several longitudinal cohort studies link fermented dairy product consumption to reduced cardiometabolic and vascular disease risk, diseases driven by pro-inflammatory immune activity (Dehghan et al., 2018, Elwood et al., 2010, Astrup, 2014, Lordan and Zabetakis, 2017, Drummond et al., 2019). Elucidating the mechanisms behind these effects by examining the interactions between fermented dairy products and cells mediating innate immunity provides insight into the potential immune impact of milk products in the diet.

Our findings show that milk fermented with *L. rhamnosus* R0011 has an impact on regulatory and pro-inflammatory cytokine production from LPS-challenged U937 and THP-1 human monocytes. When U937 monocytes were cultured with the LrF and LPS, there was a significant down-regulation of LPS-induced sCD54 production. sCD54 is a soluble form of CD54 (ICAM-1) which has been associated with increased cardiovascular disease risk and has been recently used as a marker of chronic inflammation in atherosclerotic plaques (Witkowska and Borawska, 2004). This downregulation of sCD54 was also observed for U397 monocytes incubated with the whole lactic acid control and unfermented milk controls suggesting that milk alone may exert an impact on the production of this cytokine. Conversely, there was no effect of the LrF or milk controls on LPS-induced sCD54 production by THP-1 monocytes and no modulation of LPS-induced IL-1Ra or IL-8 production by either THP-1 or U937 monocytes. The ability of unfermented milk controls to down-regulate sCD54 production on U937 monocytes is interesting in light of recent findings indicating that raw bovine milk can condition human monocytes to respond to a TLR1/2 ligand with heightened production IL-6, a response consistent with a trained immune phenotype (van Splunter et al., 2018). In keeping with these findings, we also observed upregulation of *TLR1* expression by unfermented milk controls. Bovine casein and whey hydrolysates have been shown to differentially affect TLR signaling in human macrophages (Kiewiet et al., 2017), further illustrating the potential of bovine milk components to influence macrophage activity and the complexity of milk and fermented milk products as immunomodulatory dietary components.

U937 and THP-1 monocytes were differentiated into macrophages in order to examine the effects of LrF on these key cell mediators in the innate immune response. Previous work has indicated differential effects of milk ferments on cytokine production and adhesion molecule expression by U937 cells at the monocyte and macrophage stages (Masotti et al., 2011). Interestingly, there was an increase in IL-6 production by LPS-stimulated U937 macrophages cultured with the LrF which was not observed when LPS-stimulated cells were cultured with the filtered LrF or milk controls. Further, there was a significant increase in the production of the regulatory cytokine IL-10 by LPS-stimulated U937 macrophages cultured with the LrF relative to those cells cultured with the whole lactic acid milk control and the unfermented milk control. Culturing of LPS-stimulated U937 macrophages with either the whole or filtered LrF also resulted in an increase in IL-1Ra production relative to the negative control. However, there was no modulation of sCD54, IL-8, IL-1β, TNF-α, IL-12p70 or TGF-β production from LPS-stimulated U937 macrophages cultured with the LrF or milk controls and LPS.

Similarly, the LrF had differential impacts on LPS-induced cytokine expression profiles in THP-1 macrophages. LPS-induced IL-10 and IL-1β production was significantly higher in THP-1 macrophages cultured with the LrF, an effect which was independent from acidified and unfermented milk controls. IL-10 and IL-1β production by macrophages is typical of polarization into the M2 immunoregulatory phenotype (Colin et al., 2014). These changes in cytokine production profiles were only observed in macrophages cultured with the whole LrF, and not with the filtered ferment suggesting that a direct bacteria-to-host cell interaction may be required for the up-regulation of regulatory cytokine production. Indeed, there have been studies suggesting that probiotic lactobacilli increase IL-10 and IL-6 production by murine macrophages through direct cell interactions (Morita et al., 2002). Further, both commensal and pathogenic strains of bacteria can induce cytokine production and gene transcription profiles within resting macrophages suggesting polarization towards either the M1 or M2 macrophage phenotype (Christoffersen et al., 2014, Isidro et al., 2014). For this reason, the ability of the LrF to induce the expression of other cellular markers indicative of polarization into the M2 phenotype were examined. The LrF induced the expression of *DC-SIGN* and *CD206*, markers associated with M2 activation (Genin et al., 2015; Chanput et al., 2013), and of *DUSP1,* a negative regulator of pro-inflammatory innate immunity activity (Arthur and Ley, 2013). Taken together, it appears that the LrF is polarizing LPS challenged THP-1 macrophages towards an immunoregulatory M2 phenotype.

Observed differences between THP-1 and U937 monocytes in response to culture with milk ferments and non-fermented milk controls may be explained by the tissue of origin and different maturation stage between these widely used human macrophage models. THP-1 monocytes often exhibit characteristics of less mature human peripheral blood monocyte cells while U937 monocytes are more representative of mature monocytic cells (Baek et al., 2009). Further, *in vitro* differentiation of THP-1 monocytes has been shown to induce changes in gene expression which render THP-1 macrophages more susceptible to food-derived compounds (Chanput et al., 2013). Other differences observed in the responses to the milk ferments between the two cell types could reflect the different *in vitro* differentiation protocols used in this study. PMA differentiation of THP-1 monocytes into macrophages increases NF-κB activation potentially resulting in a heightened sensitivity to stimuli (Chanput et al., 2014).

Milk is a complex fermentation substrate, and a wide range of metabolites are produced during the fermentation process. Numerous bioactive molecular components in fermented milk have been identified, with a range of bioactivities. These include casein and whey protein-derived peptides with immunomodulatory and antimicrobial activity (Fernandez and Marette, 2018, Griffiths and Tellez, 2013, Zhang et al., 2019), some of which influence macrophage activity by acting as Toll-Like Receptor agonists and antagonists (Kiewiet et al., 2017). Amino acid metabolites including tryptophan derivatives, such as kynurenine and 5-hydroxytryptophan, and bioactive amines such as γ-aminobutyric acid (GABA) and histamine are also bioactive molecules with immunomodulatory potential produced during milk fermentation (Nongonierma and FitzGerald, 2015, Pessione and Cirrincione, 2016, Beermann and Hartung, 2013, Bertazzo et al., 2016), as are conjugated linoleic acid, sphingolipids and exopolysaccharides (Lordan and Zabetakis, 2017, Viladomiu et al., 2013, Brønnum et al., 2005, Bassaganya-Riera et al., 2004, Fernandez and Marette, 2018). Given the complex molecular networks produced during milk fermentation, in addition to interactions between lactobacilli and the dairy matrix, the immunomodulatory bioactivity of fermented milk may also reflect the combined impact of different bioactive components (Burgain et al., 2014, Da Silva and Rudkowska, 2015). Future work incorporating proteomic and metabolomic approaches combined with functional assessment of immune outcomes is needed to identify and characterize the bioactive components at the molecular level and to determine whether effects are mediated by specific bioactive molecules or by synergistic interactions of multiple bioactives produced during milk fermentation.

## 5 Conclusion

The results presented here indicate that milk fermented with *L. rhamnosus* R0011 increases regulatory cytokine production by LPS-challenged U937 and THP-1 macrophages, while simultaneously up-regulating the production of certain key pro-inflammatory cytokines, a cytokine profile characteristic of polarization into the immunoregulatory M2 macrophage phenotype. Coupled with this cytokine profile, the LrF also induced the gene expression of M2 macrophage-associated markers. These findings contribute to current understanding of the potential mechanisms through which dietary dairy products influence macrophages and innate immunity. Future study is needed to identify and characterize bioactive components and to examine *in vivo* impact. Further characterization of the impact of milk fermented with *L. rhamnosus* R0011 on macrophage phenotype polarization will provide insight into mechanisms through which these types of dairy products influence health outcomes linked to immune activity.

## CRediT author statement

All authors were involved in the original design of the project or contributed to experimental design, investigation and data analysis throughout (MPJ, HJT, JLS, GE and JMG-J). MPJ and GE carried out the experiments and analysed the data. All authors were involved in preparation of the manuscript and approved the final version.

## Declaration of Competing Interest

None.

## Figures and Tables

**figs1:**
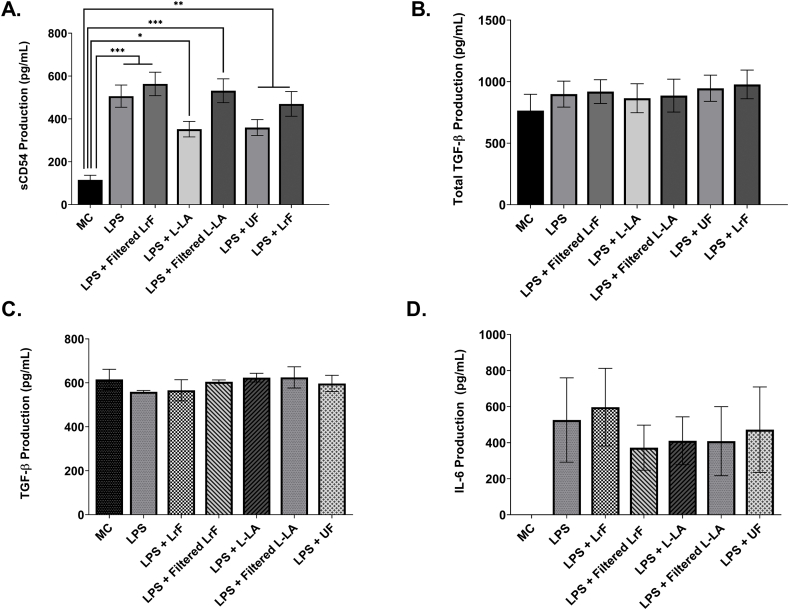
Supplemental Figure 1. U937 and THP-1 macrophages stimulated with 125 ng/mL LPS and incubated with *L. rhamnosus* R0011 whole/filtered ferments or milk controls concurrently for 24 h (n = 3) A. Mean sCD54 production from U937 macrophages ± SEM B. Mean TGF-β production ± SEM from U937 macrophages C. Mean TGF-β production from THP-1 macrophages ± SEM and D. Mean IL-6 production from THP-1 macrophages ± SEM. Cell viability was >80% for all treatments, as determined by Trypan Blue Exclusion Viability assays. Significance between treatment groups as determined by Tukey's one-way ANOVA is indicated as ∗ (P < 0.05), ∗∗ (P < 0.01), ∗∗∗ (P < 0.001), or ∗∗∗∗(P < 0.0001).

**figs2:**
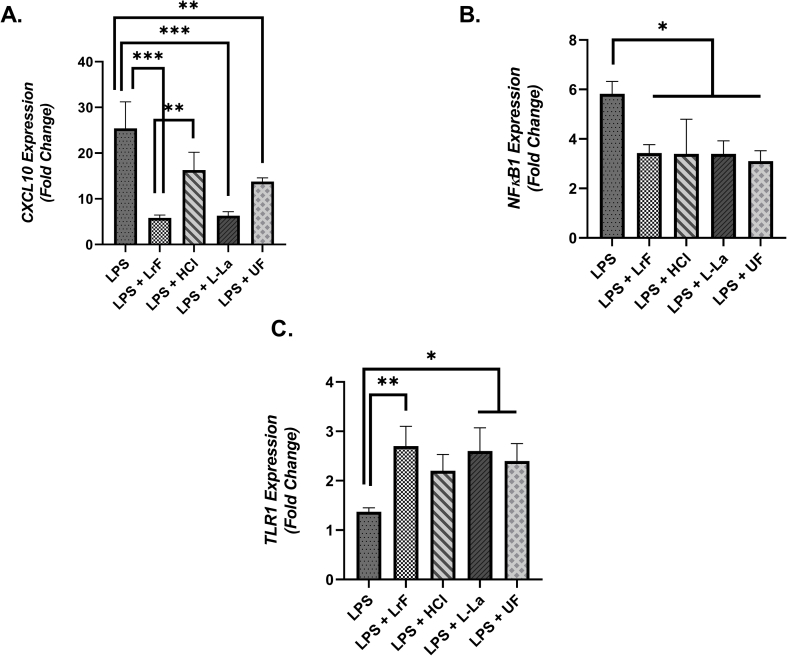
Supplemental Figure 2. Differential gene expression profiles of THP-1-derived Mθ macrophages stimulated with 125 ng/mL LPS and incubated with *L. rhamnosus* R0011 whole ferments or milk controls concurrently for 24 h (n = 3). A. Mean *CXCL10* expression ± SEM B. Mean *NFKB1* ± SEM or C. *TLR1* expression ± SEM. Significance between treatment groups as determined by Tukey's one-way ANOVA is indicated as ∗ (P < 0.05), ∗∗ (P < 0.01), ∗∗∗ (P < 0.001), or ∗∗∗∗(P < 0.0001).
