# Supplementary material for: Milk fermented with Lactobacillus rhamnosus R0011 induces a regulatory cytokine profile in LPS-challenged U937 and THP-1 macrophages
Source: Curr Res Food Sci. 2020 Feb 26;3:51–8. doi: 10.1016/j.crfs.2020.02.002 (PMC7473351; doi:10.1016/j.crfs.2020.02.002)
Supplement: Multimedia component 1 [file mmc1.docx]

**Table S1**. List of primers used for qRT-PCR in this study

| **Gene** | **Sequence** | **Reference** |
| --- | --- | --- |
| *TLR1* | F: CAGTGTCTGGTACACGCATGGT  R: TTTCAAAAACCGTGTCTGTTAAGAGA | Zarember and Godowski, 2002 |
| *DUSP1* | F: GGCCCCGAGAACAGACAAA  R: GTGCCCACTTCCATGACCAT | Locati *et al*., 2002 |
| *CXCL10* | F: TTCAAGGAGTACCTCTCTCTAG  R: CTGGATTCAGACATCTCTTCTC | MacPherson *et al*., 2014 |
| *DC-SIGN* | F: TCAAGCAGTATTGGAACAGAGGA  R: CAGGAGGCTGCGGACTTTTT | Chanput *et al.*, 2013 |
| *CD206* | F: CAGCGCTTGTGATCTTCATT  R: TACCCCTGCTCCTGGTTTTT | Chanput *et al*., 2013 |
| *NFкB1* | F: GCAGCACTACTTCTTGACCACC  R: TCTGCTCCTGAGCATTGACGTC | MacPherson *et al*., 2014 |
| *ZFP36L1* | F: ATGACCACCACCCTCGTGT  R: TTTCTGTCCAGCAGGCAACC | Chen *et al*., 2015 |
| *ActB** | F: ATTGCCGACAGGATGCAGAA  R: GCTGATCCACATCTGCTGGAA | Maeß *et al*., 2010 |
| *RPL37A** | F: ATTGAAATCAGCCAGCACGC  R: AGGAACCACAGTGCCAGATCC | Maeß *et al*., 2010 |

*Reference genes used in this study
